# The genome sequence of a longhorn beetle, *Rutpela maculata* (Poda, 1769)

**DOI:** 10.12688/wellcomeopenres.20500.2

**Published:** 2026-04-21

**Authors:** Olga Sivell, Duncan Sivell, Maxwell V. L. Barclay, Liam M. Crowley, Natural History Museum Genome Acquisition Lab, University of Oxford and Wytham Woods Genome Acquisition Lab, Darwin Tree of Life Barcoding collective, Wellcome Sanger Institute Tree of Life Management, Samples and Laboratory team, Wellcome Sanger Institute Scientific Operations: Sequencing Operations, Wellcome Sanger Institute Tree of Life Core Informatics team, Tree of Life Core Informatics collective, Darwin Tree of Life Consortium

**Affiliations:** 1 Natural History Museum, London, England, UK; 2 University of Oxford, Oxford, England, UK

**Keywords:** Rutpela maculata, longhorn beetle, genome sequence, chromosomal, Coleoptera

## Abstract

We present a genome assembly from an individual female *Rutpela maculata* (a longhorn beetle; Arthropoda; Insecta; Coleoptera; Cerambycidae). The genome sequence is 2,021.6 megabases in span. Most of the assembly is scaffolded into 10 chromosomal pseudomolecules, including the X sex chromosome. The mitochondrial genome has also been assembled and is 17.84 kilobases in length. Gene annotation of this assembly on Ensembl identified 14,238 protein-coding genes.

## Species taxonomy

Eukaryota; Metazoa; Eumetazoa; Bilateria; Protostomia; Ecdysozoa; Panarthropoda; Arthropoda; Mandibulata; Pancrustacea; Hexapoda; Insecta; Dicondylia; Pterygota; Neoptera; Endopterygota; Coleoptera; Polyphaga; Cucujiformia; Chrysomeloidea; Cerambycidae; Lepturinae; Lepturini; *Rutpela*; *Rutpela maculata* (Poda, 1761) (NCBI:txid878968).

## Background

*Rutpela maculata* is a beetle from the family Cerambycidae, commonly known as longhorn beetles. It belongs to the third-largest subfamily, Lepturinae, consisting of generally mesophilic species, usually with wood feeding larvae and diurnal flower-visiting adults, abundant in temperate forest habitats of the Northern Hemisphere, but poorly represented in the tropics, and absent from Australia.

Adults of *Rutpela maculata* are 13–20 mm in length. The pattern of black markings and spots on the yellow elytra is usually distinctive, but the markings can be variable and specimens with almost uniformly black or yellow elytra can be encountered. *Rutpela maculata* can be distinguished from similar species by the following characters: antennal segments are black with a yellow base; the legs are mostly yellow with dark tarsi, although the hind legs are variable with darker parts of tibiae and tarsi to entirely dark ( Bense, 1995). Males have the inner face of the tibiae developed into a complex structure used for grasping the female during copulation.

*Rutpela maculata* is very common and widely distributed in England and Wales, whereas in Scotland it occurs mostly in the south. Adults can be seen from May to August ( Twinn & Harding, 1999) with a peak emergence in late June and early July. The larvae feed on decaying wood, mainly of deciduous trees ( James, 2018). The life cycle takes 2 to 3 years: the beetles pupate within dead wood and the adult stage lasts 2 to 4 weeks. They are attracted to flowers on which they feed and copulate (UK Beetles, no date), and are usually seen, sometimes in numbers, in bright sunlight on umbellifers and bramble flowers at the edges of woods.

This species was previously placed in the genera *Strangalia* Dejean, 1835 and *Leptura* Linnaeus, 1758. It is widespread throughout Europe and western Asia. It has also been referred to as the spotted longhorn, the black-and-yellow longhorn ( Brock, 2021), the harlequin beetle ( Twinn & Harding, 1999) and the harlequin longhorn ( James, 2018) (though the latter two names are misleading as they are usually used for the common Neotropical longhorn *Acrocinus longimanus* (Linnaeus, 1758)). Numerous colour variations have been studied ( Uhthoff-Kaufmann, 1946) and some have been described as different subspecies ( Özdikmen *et al.*, 2012; Özdikmen, 2021). The value of describing colour varieties has been questioned, however ( James, 2018).

The high-quality genome of *Rutpela maculata* was sequenced as part of the Darwin Tree of Life Project, a collaborative effort to sequence all named eukaryotic species in the Atlantic Archipelago of Britain and Ireland. Here we present a chromosomally complete genome sequence for Rutpela maculata, based on one specimen from Luton, England.

## Genome sequence report

**Figure 1.**
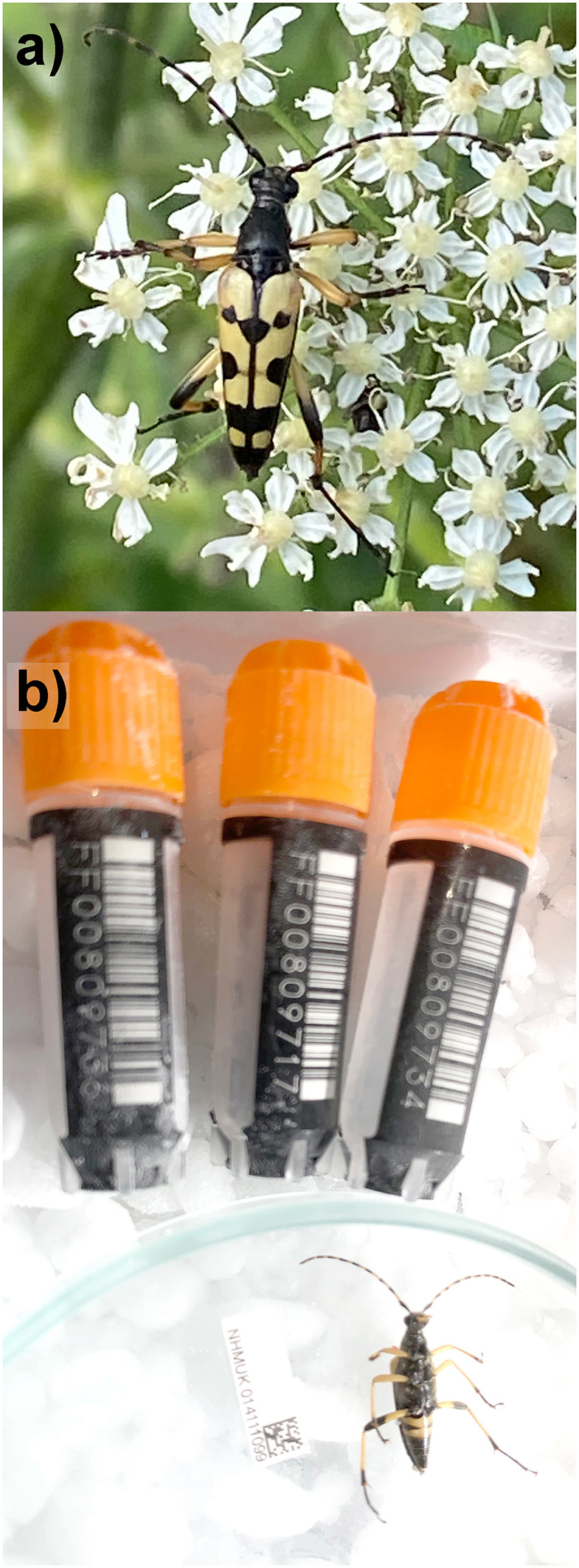
Photograph of a) Live *Rutpela maculata* b) the *Rutpela maculata* specimen (NHMUK014111099; icLepMacu1) used for genome sequencing.

The genome was sequenced from one female *Rutpela maculata* ( Figure 1) collected from Wigmore Park, Luton, UK (51.88, –0.37). A total of 32-fold coverage in Pacific Biosciences single-molecule HiFi long reads and 19-fold coverage in 10X Genomics read clouds were generated. Primary assembly contigs were scaffolded with chromosome conformation Hi-C data. Manual assembly curation corrected 180 missing joins or mis-joins and removed 15 haplotypic duplications, reducing the assembly length by 0.29% and the scaffold number by 25.23%, and increasing the scaffold N50 by 4.14%.

**Figure 2.**
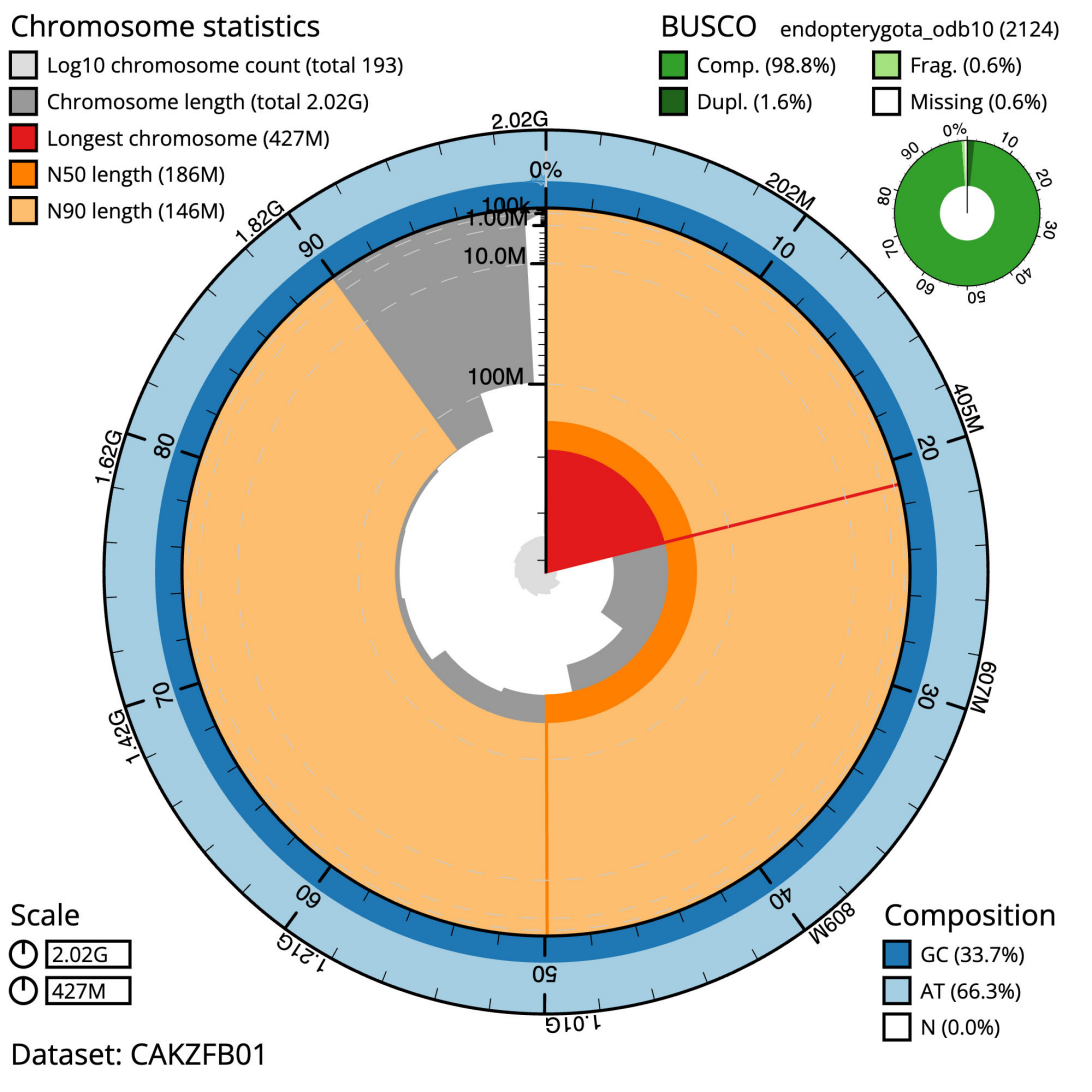
Genome assembly of *Rutpela maculata*, icLepMacu1.2: metrics. The BlobToolKit Snailplot shows N50 metrics and BUSCO gene completeness. The main plot is divided into 1,000 size-ordered bins around the circumference with each bin representing 0.1% of the 2,022,614,896 bp assembly. The distribution of scaffold lengths is shown in dark grey with the plot radius scaled to the longest scaffold present in the assembly (426,962,397 bp, shown in red). Orange and pale-orange arcs show the N50 and N90 scaffold lengths (185,716,378 and 146,097,932 bp), respectively. The pale grey spiral shows the cumulative scaffold count on a log scale with white scale lines showing successive orders of magnitude. The blue and pale-blue area around the outside of the plot shows the distribution of GC, AT and N percentages in the same bins as the inner plot. A summary of complete, fragmented, duplicated and missing BUSCO genes in the endopterygota_odb10 set is shown in the top right. An interactive version of this figure is available at https://blobtoolkit.genomehubs.org/view/icLepMacu1.1/dataset/CAKZFB01/snail.

**Figure 3.**
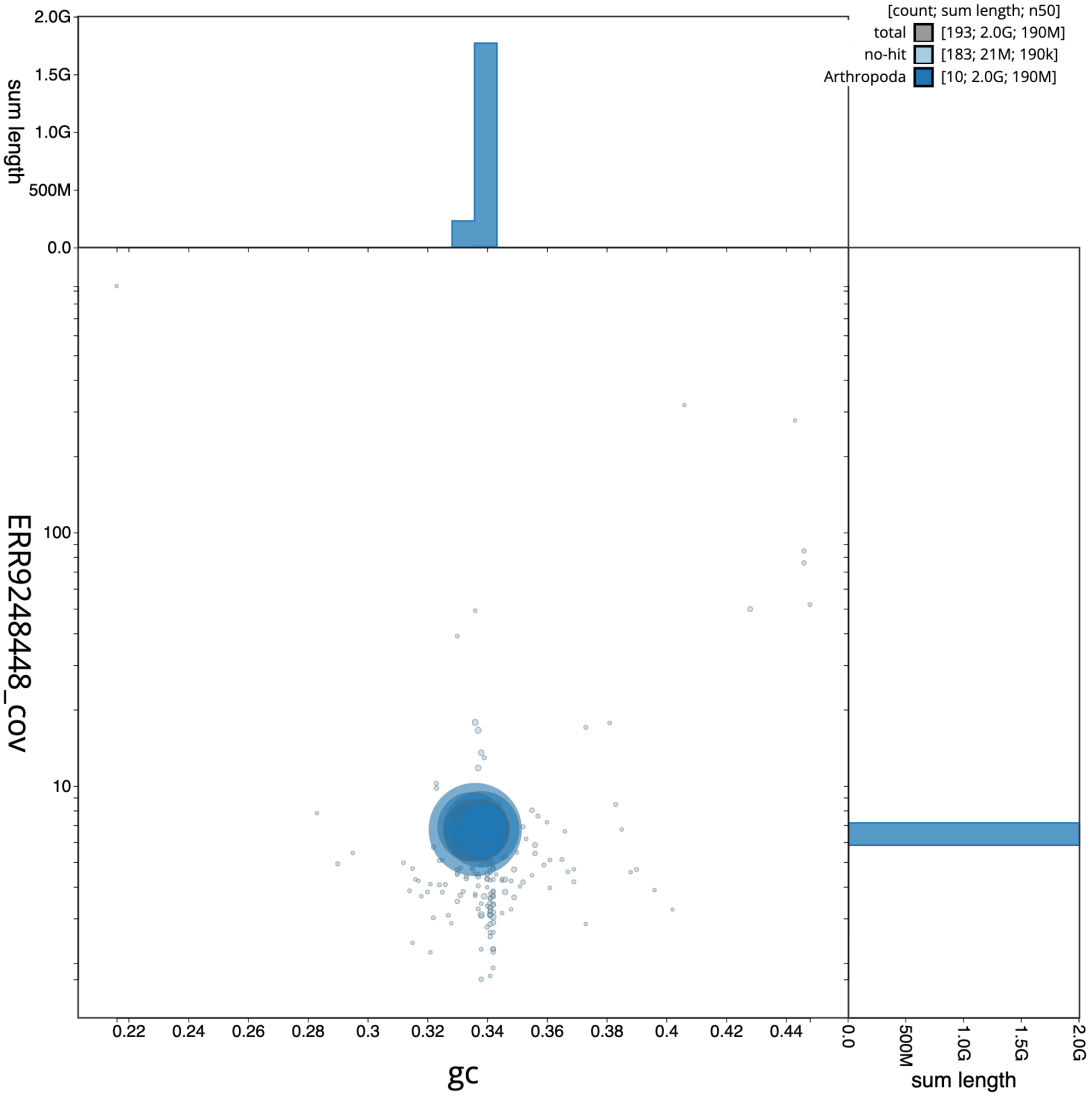
Genome assembly of *Rutpela maculata*, icLepMacu1.2: BlobToolKit GC-coverage plot. Scaffolds are coloured by phylum. Circles are sized in proportion to scaffold length. Histograms show the distribution of scaffold length sum along each axis. An interactive version of this figure is available at https://blobtoolkit.genomehubs.org/view/icLepMacu1.1/dataset/CAKZFB01/blob.

**Figure 4.**
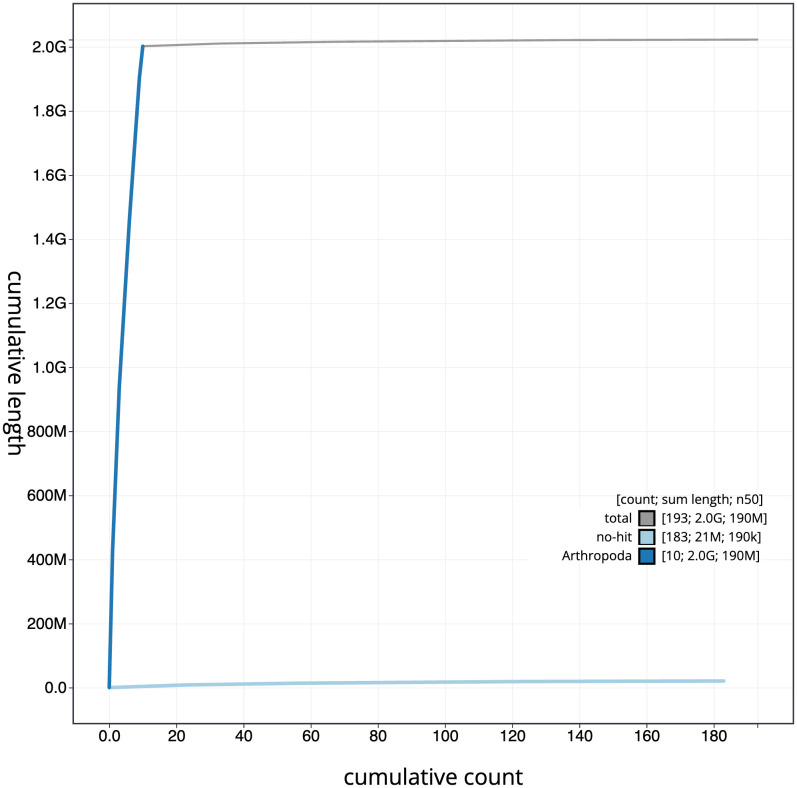
Genome assembly of *Rutpela maculata*, icLepMacu1.2: BlobToolKit cumulative sequence plot. The grey line shows cumulative length for all scaffolds. Coloured lines show cumulative lengths of scaffolds assigned to each phylum using the buscogenes taxrule. An interactive version of this figure is available at https://blobtoolkit.genomehubs.org/view/icLepMacu1.1/dataset/CAKZFB01/cumulative.

**Figure 5.**
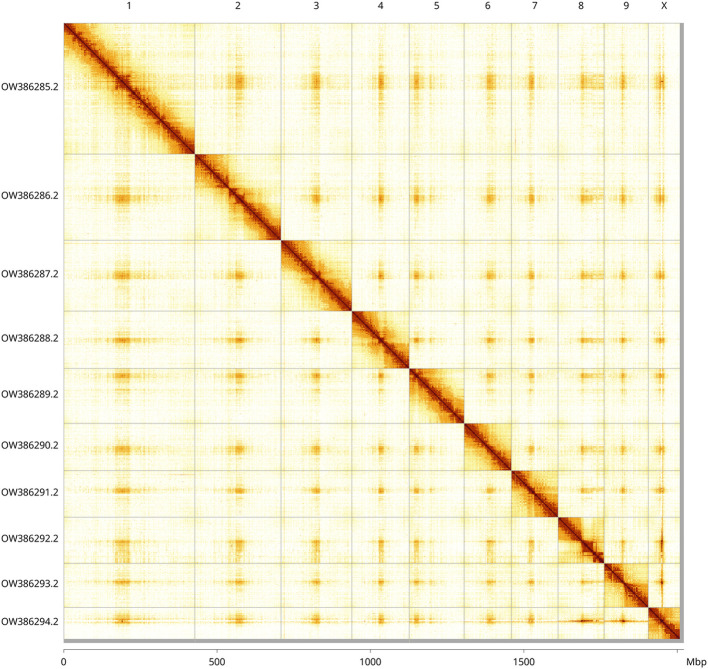
Genome assembly of *Rutpela maculata*, icLepMacu1.2: Hi-C contact map of the icLepMacu1.2 assembly, visualised using HiGlass. Chromosomes are shown in order of size from left to right and top to bottom. An interactive version of this figure may be viewed at https://genome-note-higlass.tol.sanger.ac.uk/l/?d=Ty8HhluiQB2Vp7anXTu1nw.

The final assembly has a total length of 2,021.6 Mb in 165 sequence scaffolds with a scaffold N50 of 187.0 Mb ( Table 1). A summary of the assembly statistics is shown in Figure 2, while the distribution of assembly scaffolds on GC proportion and coverage is shown in Figure 3. The cumulative assembly plot in Figure 4 shows curves for subsets of scaffolds assigned to different phyla. Most (99.38%) of the assembly sequence was assigned to 10 chromosomal-level scaffolds, representing 9 autosomes and the X sex chromosome. A summary of the assembly statistics is shown in Figure 2, while the distribution of assembly scaffolds on GC proportion and coverage is shown in Figure 3. The cumulative assembly plot in Figure 4 shows curves for subsets of scaffolds assigned to different phyla. Chromosome-scale scaffolds confirmed by the Hi-C data are named in order of size ( Figure 5; Table 2). While not fully phased, the assembly deposited is of one haplotype. Contigs corresponding to the second haplotype have also been deposited. The mitochondrial genome was also assembled and can be found as a contig within the multifasta file of the genome submission.

**Table 1.** Genome data for *Rutpela maculata*, icLepMacu1.2.

Project accession data		
Assembly identifier	icLepMacu1.2	
Species	*Rutpela maculata*	
Specimen	icLepMacu1	
NCBI taxonomy ID	878968	
BioProject	PRJEB51454	
BioSample ID	SAMEA7521529	
Isolate information	icLepMacu1, thorax (DNA sequencing and Hi-C data) icLepMacu2: abdomen (RNA sequencing)	
Assembly metrics *		***Benchmark***
Consensus quality (QV)	Primary: 56.0; alternate: 53.6; combined: 54.4	≥ *40*
*k*-mer completeness	Primary: 83.96%; alternate: 74.87%; combined: 98.49%	≥ *95%*
BUSCO **	C:98.8%[S:97.2%,D:1.6%],F:0.6%,M:0.6%,n:2,124	*C* ≥ *95%*
Percentage of assembly mapped to chromosomes	99.38%	≥ *90%*
Sex chromosomes	X chromosome	*localised homologous pairs*
Organelles	Mitochondrial genome assembled	*complete single alleles*
**Raw data accessions**		
PacificBiosciences SEQUEL II	ERR9284045, ERR9284046, ERR9284044	
Chromium 10X	ERR9248446–ERR9248449	
Hi-C Illumina	ERR9248445	
PolyA RNA-Seq Illumina	ERR10123690	
**Genome assembly**		
Assembly accession	GCA_936432065.2	
*Accession of alternate haplotype*	GCA_936443135.2	
Span (Mb)	2,021.6	
Number of contigs	1,046	
Contig N50 length (Mb)	4.3	
Number of scaffolds	165	
Scaffold N50 length (Mb)	187.0	
Longest scaffold (Mb)	427.5	

* Assembly metric benchmarks are adapted from column VGP-2020 of “ Table 1: Proposed standards and metrics for defining genome assembly quality” from ( Rhie *et al.*, 2021).

** BUSCO scores based on the endopterygota_odb10 BUSCO set using v5.3.2. C = complete [S = single copy, D = duplicated], F = fragmented, M = missing, n = number of orthologues in comparison. A full set of BUSCO scores is available at https://blobtoolkit.genomehubs.org/view/Rutpela%20maculata/dataset/CAKZFB01/busco.

**Table 2.** Chromosomal pseudomolecules in the genome assembly of *Rutpela maculata*, icLepMacu1.

INSDC accession	Chromosome	Length (Mb)	GC%
OW386285.2	1	427.5	33.5
OW386286.2	2	280.79	34.0
OW386287.2	3	231.23	33.5
OW386288.2	4	187.01	33.5
OW386289.2	5	178.95	33.5
OW386290.2	6	153.72	33.5
OW386291.2	7	152.32	34.0
OW386292.2	8	149.97	34.0
OW386293.2	9	144.23	34.0
OW386294.2	X	103.24	34.0
OW386295.2	MT	0.02	21.5

The combined primary and alternate assemblies achieve an estimated QV of 54.4. The *k*-mer completeness is 83.96% for the primary assembly, 74.87% for the alternate haplotype, and 98.49% for the combined assemblies. The primary assembly has a BUSCO v5.3.2 completeness of 98.8% (single = 97.2%, duplicated = 1.6%), using the endopterygota_odb10 reference set ( *n* = 2,124).

## Genome annotation report

The *Rutpela maculata* genome assembly (GCA_936432065.2) was annotated by Ensembl at the European Bioinformatics Institute (EBI). This annotation includes 23 845 transcribed mRNAs from 14 238 protein-coding and 1 989 non-coding genes. The average transcript length is 34 645.16 bp, with an average of 1.47 coding transcripts per gene and 5.48 exons per transcript. Further details of this annotation are available from the Ensembl annotation page.

## Methods

### Sample acquisition and nucleic acid extraction

The adult female *Rutpela maculata* specimen used for genome sequencing (specimen ID NHMUK014111099, ToLID icLepMacu1) was collected from Wigmore Park, Luton, UK (latitude 51.88, longitude –0.37) on 2020-07-07. The specimen was collected by Olga Sivell and identified by Duncan Sivell (both affiliated to the Natural History Museum) and snap-frozen on dry ice. The specimen used for RNA sequencing (Ox000477, icLepMacu2) was collected by Liam Crowley (University of Oxford) on 2020-06-15 from Wytham Woods, Oxford.

The workflow for high molecular weight (HMW) DNA extraction at the Wellcome Sanger Institute (WSI) includes a sequence of core procedures: sample preparation; sample homogenisation; DNA extraction; HMW DNA fragmentation; and fragmented DNA clean-up. The icLepMacu1 sample was weighed and dissected on dry ice with tissue set aside for Hi-C sequencing (as per the protocol at https://dx.doi.org/10.17504/protocols.io.x54v9prmqg3e/v1). For sample homogenisation, thorax tissue was cryogenically disrupted using the Sample Homogenisation: Covaris cryoPREP ® Automated Dry Pulverizer protocol ( https://dx.doi.org/10.17504/protocols.io.eq2lyjp5qlx9/v1). HMW DNA was extracted by means of the Manual MagAttract protocol ( https://dx.doi.org/10.17504/protocols.io.6qpvr33novmk/v1). HMW DNA was sheared into an average fragment size of 12–20 kb in a Megaruptor 3 system with speed setting 30, following the HMW DNA Fragmentation: Diagenode Megaruptor ®3 for PacBio HiFi protocol ( https://dx.doi.org/10.17504/ protocols.io.8epv5x2zjg1b/v1). Sheared DNA was purified by solid-phase reversible immobilisation (SPRI) (protocol at https://dx.doi.org/10.17504/protocols.io.kxygx3y1dg8j/v1). In brief, the method employs a 1.8X ratio of AMPure PB beads to sample to eliminate shorter fragments and concentrate the DNA. The concentration of the sheared and purified DNA was assessed using a Nanodrop spectrophotometer and Qubit Fluorometer and Qubit dsDNA High Sensitivity Assay kit. Fragment size distribution was evaluated by running the sample on the FemtoPulse system.

RNA was extracted from abdomen tissue of icLepMacu2 in the Tree of Life Laboratory at the WSI using the RNA Extraction: Automated MagMax™ *mir*Vana protocol ( https://dx.doi.org/10.17504/protocols.io.6qpvr36n3vmk/v1). The RNA concentration was assessed using a Nanodrop spectrophotometer and Qubit Fluorometer using the Qubit RNA Broad-Range (BR) Assay kit. Analysis of the integrity of the RNA was done using the Agilent RNA 6000 Pico Kit and Eukaryotic Total RNA assay.

Protocols employed by the Tree of Life laboratory are publicly available on protocols.io: https://dx.doi.org/10.17504/protocols.io.8epv5xxy6g1b/v1.

### Sequencing

Pacific Biosciences HiFi circular consensus and 10X Genomics read cloud DNA sequencing libraries were constructed according to the manufacturers’ instructions. Poly(A) RNA-Seq libraries were constructed using the NEB Ultra II RNA Library Prep kit. DNA and RNA sequencing were performed by the Scientific Operations core at the WSI on Pacific Biosciences SEQUEL II (HiFi), Illumina NovaSeq 6000 (RNA-Seq) and HiSeq X Ten (10X) instruments. Hi-C data were also generated from thorax tissue of icLepMacu1 using the Arima2 kit and sequenced on the Illumina NovaSeq 6000 instrument.

### Genome assembly, curation and evaluation

Assembly was carried out with Hifiasm ( Cheng *et al.*, 2021) and haplotypic duplication was identified and removed with purge_dups ( Guan *et al.*, 2020). One round of polishing was performed by aligning 10X Genomics read data to the assembly with Long Ranger ALIGN, calling variants with FreeBayes ( Garrison & Marth, 2012). The assembly was then scaffolded with Hi-C data ( Rao *et al.*, 2014) using YaHS ( Zhou *et al.*, 2023). The assembly was checked for contamination and corrected as described previously ( Howe *et al.*, 2021). Manual curation was performed using HiGlass ( Kerpedjiev *et al.*, 2018) and Pretext ( Harry, 2022). The mitochondrial genome was assembled using MitoHiFi ( Uliano-Silva *et al.*, 2022), which runs MitoFinder ( Allio *et al.*, 2020) and uses these annotations to select the final mitochondrial contig and to ensure the general quality of the sequence.

A Hi-C map for the final assembly was produced using bwa-mem2 ( Vasimuddin *et al.*, 2019) in the Cooler file format ( Abdennur & Mirny, 2020). To assess the assembly metrics, the *k*-mer completeness and QV consensus quality values were calculated in Merqury.FK ( Rhie *et al.*, 2020). The genome was analysed within the BlobToolKit environment ( Challis *et al.*, 2020) and BUSCO scores ( Manni *et al.*, 2021) were calculated.

Table 3 contains a list of relevant software tool versions and sources.

**Table 3.** Software tools: versions and sources.

Software tool	Version	Source
BlobToolKit	4.1.7	https://github.com/blobtoolkit/blobtoolkit
BUSCO	5.3.2	https://gitlab.com/ezlab/busco
FastK	1.1	https://github.com/thegenemyers/FASTK
FreeBayes	1.3.1-17-gaa2ace8	https://github.com/freebayes/freebayes
gEVAL	N/A	https://geval.org.uk/
Hifiasm	0.16.1-r375	https://github.com/chhylp123/hifiasm
HiGlass	1.11.6	https://github.com/higlass/higlass
Long Ranger ALIGN	2.2.2	https://support.10xgenomics.com/genome-exome/ software/pipelines/latest/advanced/other-pipelines
Merqury.FK	1.1.2	https://github.com/thegenemyers/MERQURY.FK
MitoHiFi	2	https://github.com/marcelauliano/MitoHiFi
PretextView	0.2.5	https://github.com/wtsi-hpag/PretextView
purge_dups	1.2.3	https://github.com/dfguan/purge_dups
YaHS	yahs-1.1.91eebc2	https://github.com/c-zhou/yahs

### Wellcome Sanger Institute – Legal and Governance

The materials that have contributed to this genome note have been supplied by a Darwin Tree of Life Partner. The submission of materials by a Darwin Tree of Life Partner is subject to the **‘Darwin Tree of Life Project Sampling Code of Practice’**, which can be found in full on the Darwin Tree of Life website here. By agreeing with and signing up to the Sampling Code of Practice, the Darwin Tree of Life Partner agrees they will meet the legal and ethical requirements and standards set out within this document in respect of all samples acquired for, and supplied to, the Darwin Tree of Life Project.

Further, the Wellcome Sanger Institute employs a process whereby due diligence is carried out proportionate to the nature of the materials themselves, and the circumstances under which they have been/are to be collected and provided for use. The purpose of this is to address and mitigate any potential legal and/or ethical implications of receipt and use of the materials as part of the research project, and to ensure that in doing so we align with best practice wherever possible. The overarching areas of consideration are:

• Ethical review of provenance and sourcing of the material
• Legality of collection, transfer and use (national and international)

Each transfer of samples is further undertaken according to a Research Collaboration Agreement or Material Transfer Agreement entered into by the Darwin Tree of Life Partner, Genome Research Limited (operating as the Wellcome Sanger Institute), and in some circumstances other Darwin Tree of Life collaborators.

## Data availability

European Nucleotide Archive: *Rutpela maculata.* Accession number PRJEB51454; https://identifiers.org/ena.embl/PRJEB51454. The genome sequence is released openly for reuse. The *Rutpela maculata* genome sequencing initiative is part of the Darwin Tree of Life (DToL) project. All raw sequence data and the assembly have been deposited in INSDC databases. Raw data and assembly accession identifiers are reported in Table 1.

Production code used in genome assembly at the WSI Tree of Life is available at https://github.com/sanger-tol .

## Author information

Members of the Natural History Museum Genome Acquisition Lab are listed here: https://doi.org/10.5281/zenodo.4790042.

Members of the University of Oxford and Wytham Woods Genome Acquisition Lab are listed here: https://doi.org/10.5281/zenodo.7125292.

Members of the Darwin Tree of Life Barcoding collective are listed here: https://doi.org/10.5281/zenodo.4893703.

Members of the Wellcome Sanger Institute Tree of Life Management, Samples and Laboratory team are listed here: https://doi.org/10.5281/zenodo.10066175.

Members of Wellcome Sanger Institute Scientific Operations: Sequencing Operations are listed here: https://doi.org/10.5281/zenodo.10043364.

Members of the Wellcome Sanger Institute Tree of Life Core Informatics team are listed here: https://doi.org/10.5281/zenodo.10066637.

Members of the Tree of Life Core Informatics collective are listed here: https://doi.org/10.5281/zenodo.5013541.

Members of the Darwin Tree of Life Consortium are listed here: https://doi.org/10.5281/zenodo.4783558.

## References

[ref1] Abdennur N Mirny LA : Cooler: Scalable storage for Hi-C data and other genomically labeled arrays. *Bioinformatics.* 2020;36(1):311–316. 10.1093/bioinformatics/btz540 31290943 PMC8205516

[ref2] Allio R Schomaker-Bastos A Romiguier J : MitoFinder: Efficient automated large-scale extraction of mitogenomic data in target enrichment phylogenomics. *Mol Ecol Resour.* 2020;20(4):892–905. 10.1111/1755-0998.13160 32243090 PMC7497042

[ref3] Bense U : Longhorn beetles. Illustrated key to the Cerambycidae and Vesperidae of Europe. Weikersheim: Margraf Verlag;1995. Reference Source

[ref4] Brock PD : Britain’s Insects. A field guide to the insects of Great Britain and Ireland. Princeton, NJ: WildGuides, Princeton University Press,2021. 10.2307/j.ctvrf8b4g Reference Source

[ref5] Challis R Richards E Rajan J : BlobToolKit - interactive quality assessment of genome assemblies. *G3 (Bethesda).* 2020;10(4):1361–1374. 10.1534/g3.119.400908 32071071 PMC7144090

[ref6] Cheng H Concepcion GT Feng X : Haplotype-resolved *de novo* assembly using phased assembly graphs with hifiasm. *Nat Methods.* 2021;18(2):170–175. 10.1038/s41592-020-01056-5 33526886 PMC7961889

[ref7] Garrison E Marth G : Haplotype-based variant detection from short-read sequencing. 2012; (Accessed: 26 July 2023). 10.48550/arXiv.1207.3907

[ref8] Guan D McCarthy SA Wood J : Identifying and removing haplotypic duplication in primary genome assemblies. *Bioinformatics.* 2020;36(9):2896–2898. 10.1093/bioinformatics/btaa025 31971576 PMC7203741

[ref9] Harry E : PretextView (Paired REad TEXTure Viewer): A desktop application for viewing pretext contact maps. 2022; (Accessed: 19 October 2022). Reference Source

[ref10] Howe K Chow W Collins J : Significantly improving the quality of genome assemblies through curation. *GigaScience* *.* Oxford University Press,2021;10(1): giaa153. 10.1093/gigascience/giaa153 33420778 PMC7794651

[ref11] James TJ : *Beetles of Hertfordshire.* Hertfordshire Natural History Society;2018.

[ref12] Kerpedjiev P Abdennur N Lekschas F : HiGlass: web-based visual exploration and analysis of genome interaction maps. *Genome Biol.* 2018;19(1):125. 10.1186/s13059-018-1486-1 30143029 PMC6109259

[ref13] Manni M Berkeley MR Seppey M : BUSCO update: Novel and streamlined workflows along with broader and deeper phylogenetic coverage for scoring of eukaryotic, prokaryotic, and viral genomes. *Mol Biol Evol.* 2021;38(10):4647–4654. 10.1093/molbev/msab199 34320186 PMC8476166

[ref14] Özdikmen H Mercan N Cihan N : Subspecific status of Rutpela maculata (Poda, 1761) (Coleoptera: Cerambycidae: Lepturinae). *Mun Ent Zool.* 2012;7(1):516–522. Reference Source

[ref15] Özdikmen H : A discussion on taxonomic position of *Rutpela maculata manca* (Schaufuss, 1863) (Cerambycidae: Lepturinae: Lepturini). *Mun Ent Zool.* 2021;16(1):411–418. Reference Source

[ref16] Rao SSP Huntley MH Durand NC : A 3D map of the human genome at kilobase resolution reveals principles of chromatin looping. *Cell.* 2014;159(7):1665–1680. 10.1016/j.cell.2014.11.021 25497547 PMC5635824

[ref17] Rhie A McCarthy SA Fedrigo O : Towards complete and error-free genome assemblies of all vertebrate species. *Nature.* 2021;592(7856):737–746. 10.1038/s41586-021-03451-0 33911273 PMC8081667

[ref18] Rhie A Walenz BP Koren S : Merqury: Reference-free quality, completeness, and phasing assessment for genome assemblies. *Genome Biology.* 2020;21(1):245. 10.1186/s13059-020-02134-9 32928274 PMC7488777

[ref19] Twinn PFG Harding PT : *Provisional atlas of the longhorn beetles (Coleoptera, Cerambycidae) of Britain.* Huntingdon: Biological Records Centre,1999. Reference Source

[ref20] Uhthoff-Kaufmann RR : Preliminary notes on *Strangalia maculata* Poda (Col., Cerambycidae) and its aberrrations in Great Britain. *Entomologist’s Monthly Magazine.* 1946;82:115–117.

[ref21] UK Beetles : *Rutpela maculata* (Poda, 1761). *ukbeetles.co.uk.* (no date); (Accessed: 9 September 2023). Reference Source

[ref22] Uliano-Silva M Ferreira JGRN Krasheninnikova K : MitoHiFi: a python pipeline for mitochondrial genome assembly from PacBio High Fidelity reads. *bioRxiv.* [Preprint]. 2022. 10.1101/2022.12.23.521667 PMC10354987 37464285

[ref23] Vasimuddin M Misra S Li H : Efficient Architecture-Aware Acceleration of BWA-MEM for Multicore Systems. In: *2019 IEEE International Parallel and Distributed Processing Symposium (IPDPS).* IEEE,2019;314–324. 10.1109/IPDPS.2019.00041

[ref24] Zhou C McCarthy SA Durbin R : YaHS: yet another Hi-C scaffolding tool. *Bioinformatics.* Edited by Alkan C ,2023;39(1): btac808. 10.1093/bioinformatics/btac808 PMC9848053 36525368

