# Supplementary material for: The genome sequence of a longhorn beetle, Rutpela maculata (Poda, 1769)
Source: Wellcome Open Res. 2026 Apr 21;8:579. Originally published 2023 Dec 20. [Version 2] doi: 10.12688/wellcomeopenres.20500.2 (PMC13107107; doi:10.12688/wellcomeopenres.20500.2)
Supplement: Peer review history

# Reviewer report: Reviewer response for version 2

**DOI:** 10.21956/wellcomeopenres.28655.r153827

**Published:** 2026-04-23

**Authors:** Sean D Schoville

**Affiliations:** 1 Department of Entomology, University of Wisconsin–Madison, Madison, USA

The authors addressed my concerns and have provided a valuable resource for the research community.

Are sufficient details of methods and materials provided to allow replication by others?

Yes

Is the rationale for creating the dataset(s) clearly described?

Yes

Are the datasets clearly presented in a useable and accessible format?

Yes

Are the protocols appropriate and is the work technically sound?

Yes

Reviewer Expertise:

Evolutionary genomics of insects.

I confirm that I have read this submission and believe that I have an appropriate level of expertise to confirm that it is of an acceptable scientific standard.

# Reviewer report: Reviewer response for version 1

**DOI:** 10.21956/wellcomeopenres.22693.r99134

**Published:** 2024-09-17

**Authors:** Sean D Schoville

**Affiliations:** 1 Department of Entomology, University of Wisconsin–Madison, Madison, USA

The article by Sivell and colleagues presents a new genome assembly for the longhorn beetle, *Rutpela maculata*. This is a large genome, but it appears to be robustly assembled to a set of chromosomes with high coverage HiFi data and Hi-C data. This resulting genome will be useful for comparative genomics research in the beetle (and insect) tree of life.

**Major revision:**

 Why were RNA data were collected, if BRAKER was run in protein mode and without this input data? It would likely be better to include the RNA-seq data in the genome annotation. Perhaps this section simply needs to be revised to provide full details of what was done in this genome project.

**Minor revisions:**

- Revise this sentence: "England and Wales, whereas in Scotland it occurs mostly in the south"
- Revise this sentence: "The specimen used for genome sequencing was a *Rutpela maculata* adult female (specimen ID NHMUK014111099, ToLID icLepMacu1), collected from ..."
- Revise this sentence: "(both working in the Natural History Museum)", or remove, as your affiliations are already stated.

Are sufficient details of methods and materials provided to allow replication by others?

Yes

Is the rationale for creating the dataset(s) clearly described?

Yes

Are the datasets clearly presented in a useable and accessible format?

Yes

Are the protocols appropriate and is the work technically sound?

Yes

Reviewer Expertise:

Evolutionary genomics of insects.

I confirm that I have read this submission and believe that I have an appropriate level of expertise to confirm that it is of an acceptable scientific standard, however I have significant reservations, as outlined above.

# Response

**Published:** 2026-02-04

**Authors:** Tree of Life Team Sanger

**Affiliations:** Wellcome Trust Sanger Institute, UK

Thank you for reviewing this data note. Regarding genome annotation, annotation for this assembly was not generated by the Wellcome Sanger Institute, but by Ensembl at the European Bioinformatics Institute. We therefore did not control the choice of inputs or annotation mode used in the initial draft annotation. Since submission of the original version of the data note, the draft annotation has been superseded by an annotation produced using the Ensembl Genebuild pipeline. A link to this updated annotation has now been added to the revised manuscript, and the text has been clarified accordingly to reflect what was done in this genome project. We have made the recommended minor textual revisions to the sentences highlighted by the reviewer. Tree of Life team, Wellcome Sanger Institute

# Reviewer report: Reviewer response for version 1

**DOI:** 10.21956/wellcomeopenres.22693.r86125

**Published:** 2024-06-17

**Authors:** Liang Lü

**Affiliations:** 1 Hebei Normal University, Shijiazhuang, China

I have read the main text of the manuscript, which reports a recently described longhorn beetle, Rutpela maculata (Poda, 1769). It is a formulaic paper that publishes an NGS sequence with detailed description of the background of the sample information in taxonomy, biology, collection, etc. The authors kindly presented in this manuscript clear images that make their identification trustable. The workflows of sample preparation, DNA extraction, sequencing, assembly, and annotation are fine and aligned with their previous work/publications. So I suggest the author should consider to publish more genomes or a package of such work in a single paper or a small number of papers, because most of the information displayed in this sort of papers can be formatted. In terms of scientific contribution, a special article for a single genome looks like a storm in a teacup. Just think about that.

Are sufficient details of methods and materials provided to allow replication by others?

Yes

Is the rationale for creating the dataset(s) clearly described?

Yes

Are the datasets clearly presented in a useable and accessible format?

Yes

Are the protocols appropriate and is the work technically sound?

Yes

Reviewer Expertise:

beetle diversity and evolution

I confirm that I have read this submission and believe that I have an appropriate level of expertise to confirm that it is of an acceptable scientific standard.
